# Developing an Inpatient Electronic Medical Record Phenotype for Hospital-Acquired Pressure Injuries: Case Study Using Natural Language Processing Models

**DOI:** 10.2196/41264

**Published:** 2023-03-08

**Authors:** Elvira Nurmambetova, Jie Pan, Zilong Zhang, Guosong Wu, Seungwon Lee, Danielle A Southern, Elliot A Martin, Chester Ho, Yuan Xu, Cathy A Eastwood

**Affiliations:** 1 Centre for Health Informatics Cumming School of Medicine University of Calgary Calgary, AB Canada; 2 Department of Community Health Sciences Cumming School of Medicine University of Calgary Calgary, AB Canada; 3 Alberta Health Services Edmonton, AB Canada; 4 Department of Medicine Faculty of Medicine & Dentistry University of Alberta Edmonton, AB Canada; 5 Department of Oncology University of Calgary Tom Baker Cancer Centre Calgary, AB Canada; 6 Department of Surgery Foothills Medical Centre University of Calgary Calgary, AB Canada

**Keywords:** pressure injury, natural language processing, NLP, algorithm, phenotype algorithm, phenotyping algorithm, machine learning, electronic medical record, EMR, pressure sore, pressure wound, pressure ulcer, pressure injuries, detect

## Abstract

**Background:**

Surveillance of hospital-acquired pressure injuries (HAPI) is often suboptimal when relying on administrative health data, as International Classification of Diseases (ICD) codes are known to have long delays and are undercoded. We leveraged natural language processing (NLP) applications on free-text notes, particularly the inpatient nursing notes, from electronic medical records (EMRs), to more accurately and timely identify HAPIs.

**Objective:**

This study aimed to show that EMR-based phenotyping algorithms are more fitted to detect HAPIs than ICD-10-CA algorithms alone, while the clinical logs are recorded with higher accuracy via NLP using nursing notes.

**Methods:**

Patients with HAPIs were identified from head-to-toe skin assessments in a local tertiary acute care hospital during a clinical trial that took place from 2015 to 2018 in Calgary, Alberta, Canada. Clinical notes documented during the trial were extracted from the EMR database after the linkage with the discharge abstract database. Different combinations of several types of clinical notes were processed by sequential forward selection during the model development. Text classification algorithms for HAPI detection were developed using random forest (RF), extreme gradient boosting (XGBoost), and deep learning models. The classification threshold was tuned to enable the model to achieve similar specificity to an ICD-based phenotyping study. Each model’s performance was assessed, and comparisons were made between the metrics, including sensitivity, positive predictive value, negative predictive value, and *F*_1_-score.

**Results:**

Data from 280 eligible patients were used in this study, among whom 97 patients had HAPIs during the trial. RF was the optimal performing model with a sensitivity of 0.464 (95% CI 0.365-0.563), specificity of 0.984 (95% CI 0.965-1.000), and *F*_1_-score of 0.612 (95% CI of 0.473-0.751). The machine learning (ML) model reached higher sensitivity without sacrificing much specificity compared to the previously reported performance of ICD-based algorithms.

**Conclusions:**

The EMR-based NLP phenotyping algorithms demonstrated improved performance in HAPI case detection over ICD-10-CA codes alone. Daily generated nursing notes in EMRs are a valuable data resource for ML models to accurately detect adverse events. The study contributes to enhancing automated health care quality and safety surveillance.

## Introduction

Pressure injury (PI), also known as a pressure ulcer, is an injury of the skin and deep tissues caused by external pressures. Annually, PIs affect approximately 250,000 to 500,000 Canadians, with an estimated prevalence of 26.0% in health care institutions [1,2]. Hospital-acquired pressure injuries (HAPIs) are PIs developed during an inpatient hospital stay. HAPIs can significantly extend a patient’s hospitalization length of stay and cause severe secondary complications, such as muscle and profound tissue impairment [3]. HAPI is considered mostly preventable, and its prevalence has been reckoned as an acceptable indicator of the quality of care [4,5]. Collecting HAPI status using chart review is time and labor-intensive, thereby not suitable for large-scale population-based applications. Considering all the factors, there is a need for automated ways to accurately and timely identify HAPIs for analyzing large cohort studies that support quality improvement efforts and assisting unit managers with developing reliable patient safety programs. The International Classification of Diseases, 10th Revision, adapted to the Canadian health system (ICD-10-CA), can be used to estimate the prevalence of adverse events from administrative data. However, the coded administrative data are prone to miss positive cases: previous research demonstrated that the sensitivity of the ICD algorithm for identifying HAPI cases is around 30% compared to chart review [1]. In addition to the sensitivity issue, ICD codes are not generally assigned with a specific time when diseases occur. Therefore, they are unsuitable for reporting the time when HAPIs occur [6]. Thus, there is a need for more accurate HAPI detection.

Electronic medical records (EMRs) are used to track and organize patient information for efficient treatment of medical conditions in a secure system [7]. Free-text clinical notes in EMRs consist of detailed descriptions of patients' conditions and treatment. Additionally, clinical notes are typically written in a continuous manner across patients' interactions with health care systems, making clinical notes more real-time compared to diagnosis codes. Despite the rich information the clinical record may have, coders often cannot read every entry, given their limited time per chart and many patients have prolonged hospital stays. Recent studies suggest that using free-text in EMRs alone, or incorporating EMR data elements, can significantly improve the accuracy of case identification of specific comorbidities [8-16]. Xu et al compared the ICD algorithm with algorithms based on EMR keyword search, which achieved a high sensitivity of 0.655 (95% CI 0.601-0.710) [8]. The Canadian health system operates as a publicly funded single-payer insurance system by the federal, provincial, and territorial governments [17]. Additional crown institutions at the provincial and federal-level monitor adverse events such as HAPI. For example, in Alberta, Canadian Institute for Health Information, the federal crown corporation, works with Alberta Health Services (provincial health care agency) to monitor PIs [18,19]. To date, there is no mandatory collection of PIs within Canadian acute-care facilities. Real-time PI evaluation and auditing using ICD codes are not possible as Canadian health data systems are set up such that ICD codes are assigned outside of providing care and have a few months lag in data extraction, transfer, and load [20]. Consequently, these agencies aim to monitor but are unable to conduct real-time auditing of PIs in Canada. Therefore, there is a need to develop EMR data-specific algorithm for identifying PIs for monitoring and auditing within Canadian acute-care facilities. Our objective was to create EMR data-specific algorithms for HAPIs. Availability and implementation of PI-specific algorithms within a clinical information system would allow the abovementioned federal and provincial agencies to conduct real-time surveillance of HAPIs, improving patient safety, enhancing the quality of care, and reducing the burden of costs associated with adverse events. The EMR phenotype case detection is evaluated via comparison with confirmed HAPIs status acquired in a clinical trial [21].

## Methods

### Study Design

This is an EMR phenotyping study for enhancing HAPI identification using free-text notes. Obtained clinical trial data were linked to administrative and EMR data for model development and validation. The natural language processing (NLP) method’s performance was compared with results from the ICD validation study conducted in Alberta, Canada, by Wong et al [21]. Detailed information for HAPIs identification can be found in their study.

### Clinical Trial Data

**Figure 1:**
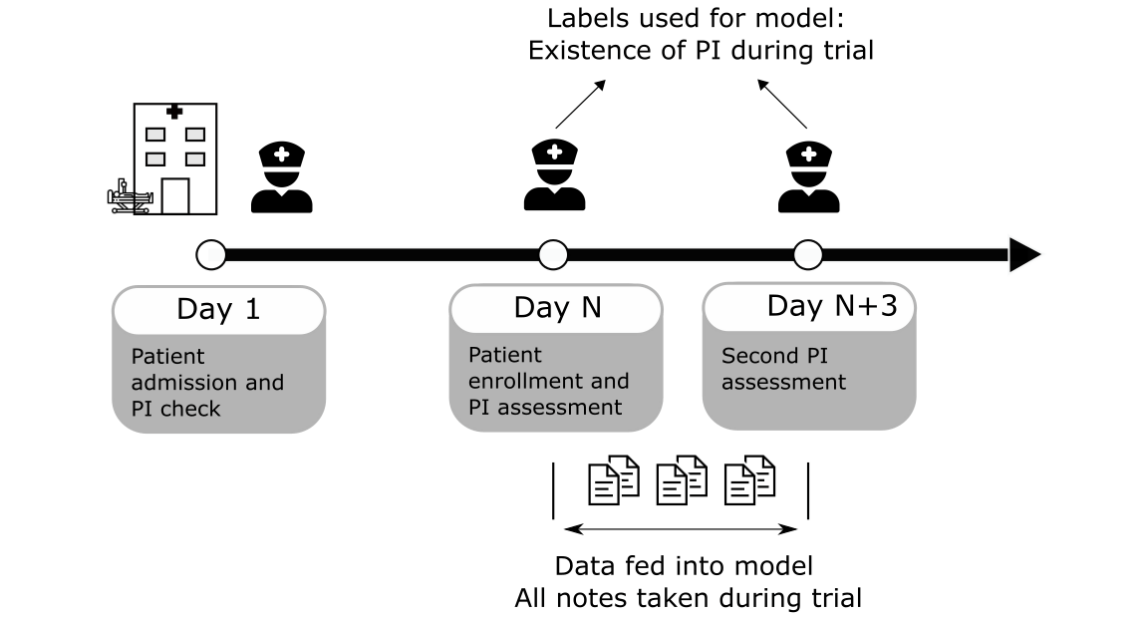
Illustration of the clinical trial for assessment of PI status in the enrolled patient cohort (n=678) and the data input used for the development of classification models. PI: pressure injury.

Previously completed randomized controlled trial (RCT) data of 678 eligible consenting inpatients were obtained from an affiliated research team and were used as the reference standard [21]. The trial evaluated the efficacy of a pressure-sensing mattress in preventing interface pressure. A research nurse performed a clinical head-to-toe skin assessment for PI formation, and suspected deep tissue injuries were monitored throughout 3 days of enrollment [21]. Assessments were conducted within 24 hours of admission, on the day of trial enrollment, and the third day after enrollment, and documented in Allscripts Sunrise Clinical Manager (SCM) EMR (Figure 1). Three days were chosen as a length of time for the research nurse to perform data collection and risk assessment for 3 reasons. First, this is the average length of stay in the local inpatient units, and a longer trial period may include varying nursing practice due to hospital discharge with a shorter length of stay, unit changes, and more nursing shift changes. Second, the dedicated investigation team deemed 3 days sufficient for pressure-related skin and soft tissue changes to develop. Lastly, as a continuous collection of interface pressure throughout the enrollment period leads to a large volume of data, 3 days allowed for optimal data collection while maintaining participant enrollment.

The research nurse, who measured pressure-related skin ulcerations, was trained as a wound care specialist in the provision of pressure ulcers, ostomy, and continence care [21]. The patients’ PI status check on admission was determined based on when the patient was admitted. The clinical trial team relied on the medical record if the patient had been admitted long before the study and consented to the study. If the patient agreed to participate in the trial right after being admitted to the hospital, the research nurse noted the PI status on admission.

The following data elements were abstracted from the clinical trial data: record ID, medical unit, sex, first-skin assessment date, second-skin assessment date, presence of PIs, and other possible related conditions (cerebrovascular disease, diabetes mellitus, etc). The clinical trial measured and classified PIs into 6 stages: stage 1, stage 2, stage 3, stage 4, suspected deep tissue injury, and unstageable PIs [22]. Stages of PIs were identified according to the National Pressure Ulcer Advisory Panel’s pressure ulcer staging system [23]. Stage 1 PIs include sores. Stage 2 captures open wounds on the surface of the skin. Stage 3 PIs represent wounds extending beneath the skin and affecting fat tissue. At stage 4, PIs are deep and reach into muscles, bones, and tendons. The trial is registered at clinicaltrials.gov (NCT02325388). Additional details surrounding the clinical trial data were published by Wong et al [21].

### Study Cohort

#### Inclusion and Exclusion Criteria

**Figure 2:**
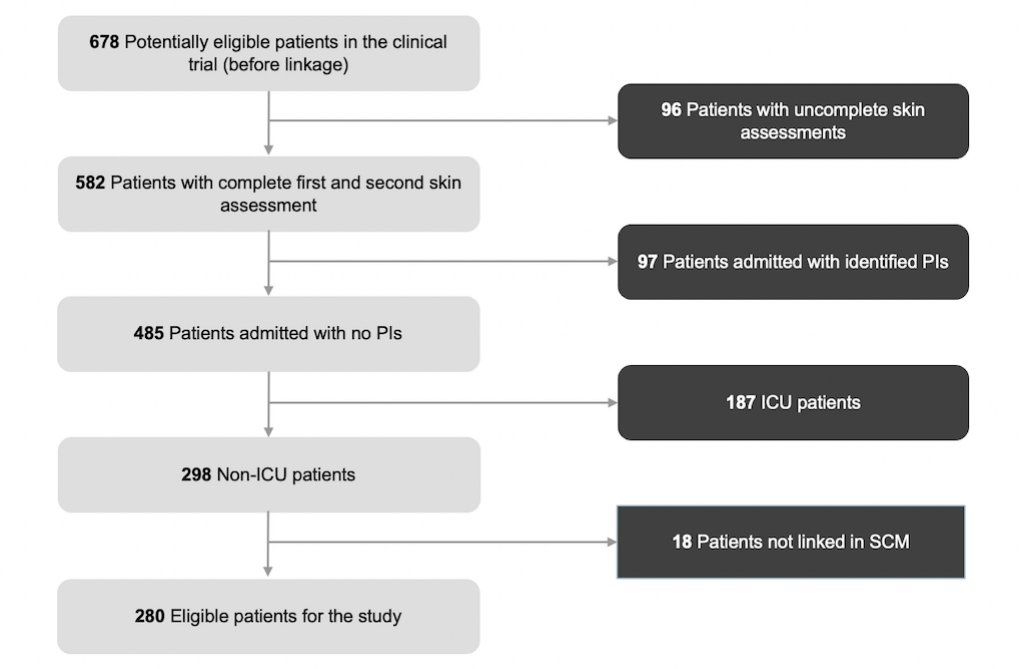
Flowchart of inclusion and exclusion criteria for the patient cohort based on trial completeness, PI status, and age (minimum age of 18 years old) for all controls in the panel. ICU: intensive care unit; PI: pressure injury; SCM: Sunrise Clinical Management.

During the RCT, eligible patients were at least 18 years old, were expected to have a length of stay of at least 3 days, and did not receive near-end-of-life care within 3 days of trial enrollment [21]. Participants were recruited from nursing units with a high risk for PI development including acute medical, neurosurgery, neurology, and intensive care [21]. For this study, patients were excluded if their data did not link to EMR data, had incomplete skin assessments, or included erroneous assessment or discharge dates. Patients with PIs on the day of admission were also excluded in order to track only PIs developed during hospitalization. Furthermore, intensive care unit (ICU) patients were excluded since their data were stored in another data warehouse with distinct data elements from those found in SCM and required restricted access. After careful selection, the final cohort of eligible patients was 280 (Figure 2).

### Data Linkage to Discharge Abstract Database and SCM EMR

Deterministic data linkage was performed between the RCT data, administrative data from the discharge abstract database (DAD), and SCM EMR data [24]. SCM was the EMR system employed in Calgary hospitals at the time of the study. Data linkage steps followed a previously established methodology [25]. First, the PI RCT data were linked to the DAD using the provincial health number and admission date. Then, DAD variables were used to connect these data with SCM.

### Document Types and Sequential Forward Type Feature Selection

In total, 37 types of documents were noted for the included patients during the clinical trial. Nursing notes were the primary source of suitable HAPI information and constituted the largest proportion of the documents. Among the nursing notes, “Patient Assessment” contained the assessment of skin and wounds under the Integument section. The Integument section described skin integrity, bruises, wound formation, and exposure to air. The “Patient Assessment Neuro” document included the patient's neurological state, where the main components related to PIs were level of consciousness, communication, and sensory deficit. The “Patient Care” document included patients' hygiene, activity, exercise, and nutrition, such as mobility, positioning, and assistance with a meal. The remaining document types contained daily intake and output, physiological indicators, pain scale, and other related data. Discharge summaries, unit transfer notes, and inpatient triage reports were not written for most patients during the clinical trial because the trial was primarily conducted in the middle of the hospital stay.

**Figure 3:**
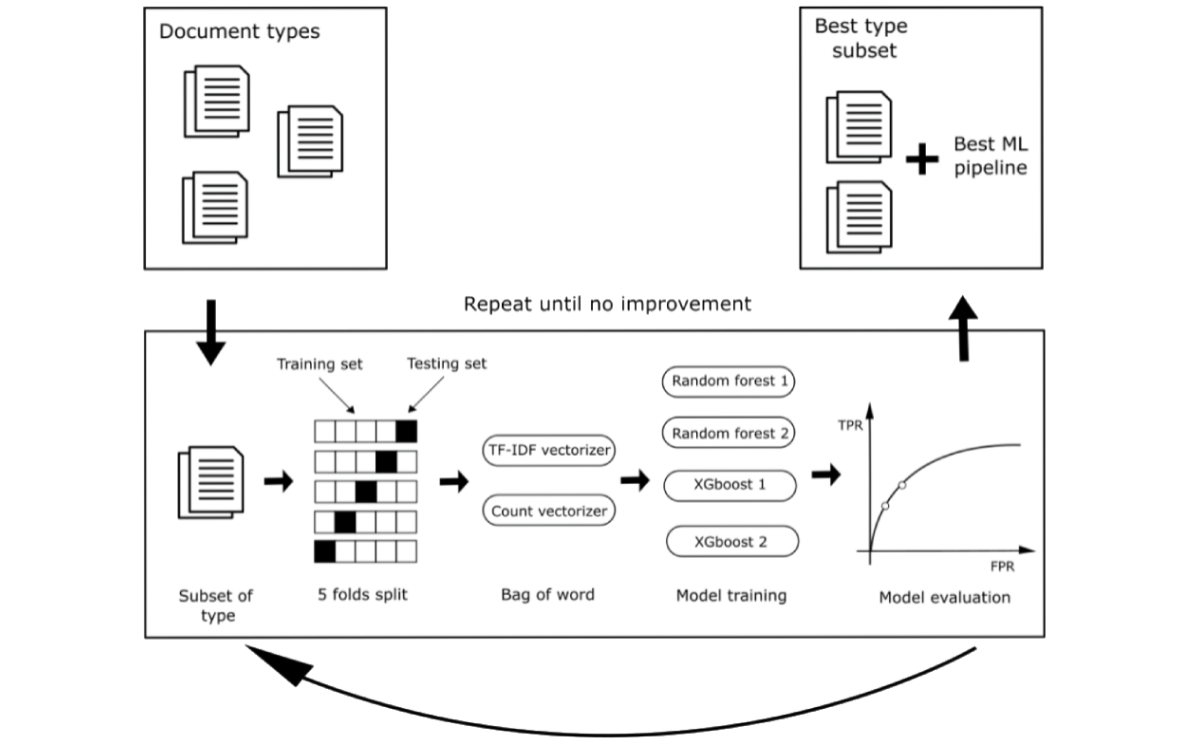
A visual illustration of the sequential forward selection process for identifying feature subsets that maximize the performance of the ML pipeline. The candidate feature subsets were evaluated by using 5-fold cross-validation. For each subset of document types, 40 experiments were conducted with all possible combinations of 5 folds, 2 vectorizers, and 4 ML models. FPR: false positive rate. ML: machine learning; TF-IDF: term frequency-inverse document frequency; TPR: true positive rate.

Forward feature selection was used to determine the best combination of documents with 2 machine learning (ML) models: extreme gradient boosting (XGBoost) and random forest (RF) [26,27]. Forward feature selection is an iterative way to obtain the best subset of features [28]. The analyses began with no feature in the input of models. Then, in each iteration, new features were added and observed for improvements (Figure 3). The experiments were run with each feature from the list of all possible features, where the best predictor was then added to our feature set. This iteration ended when introducing a new feature did not significantly improve the targeted metric. In our experiments, the forward feature selection was performed for every document type. Instead of adding 1 feature in each iteration, all documents belonging to 1 type were added to the input of models. This feature selection stopped when adding a new document type did not increase the target metric. Due to the long convergence time, the forward feature selection was not conducted during the development of the deep learning model. Rather, the same optimal document set determined by ML models was used.

### Natural Language Processing

#### Bag of Words Preprocessing and ML

All nursing notes of selected document types were merged into 1 text and converted into a bag-of-words (BOW) vector with the count of words or term frequency-inverse document frequency (TF-IDF) vectorizer by using a Python scikit-learn ML library [29-31].

A binary classification model was developed to identify HAPI cases by considering all patients who developed any stage of PI during a hospital stay as positive cases and patients without PIs as the negative cohort. The BOW matrices were used as the independent input for the models. RF and XGBoost classification models were trained to perform classification. These 2 models were chosen because they were representative of ensemble models: RF for bagging and XGboost for boosting. Ensemble models have been shown to display superior performance than a single classifier [32]. Two sets of hyperparameters were tried for each model. The 5-fold cross-validation was conducted to determine the most useful document types, high-performing ML model, and its hyperparameters.

#### Deep Learning Model

**Figure 4:**
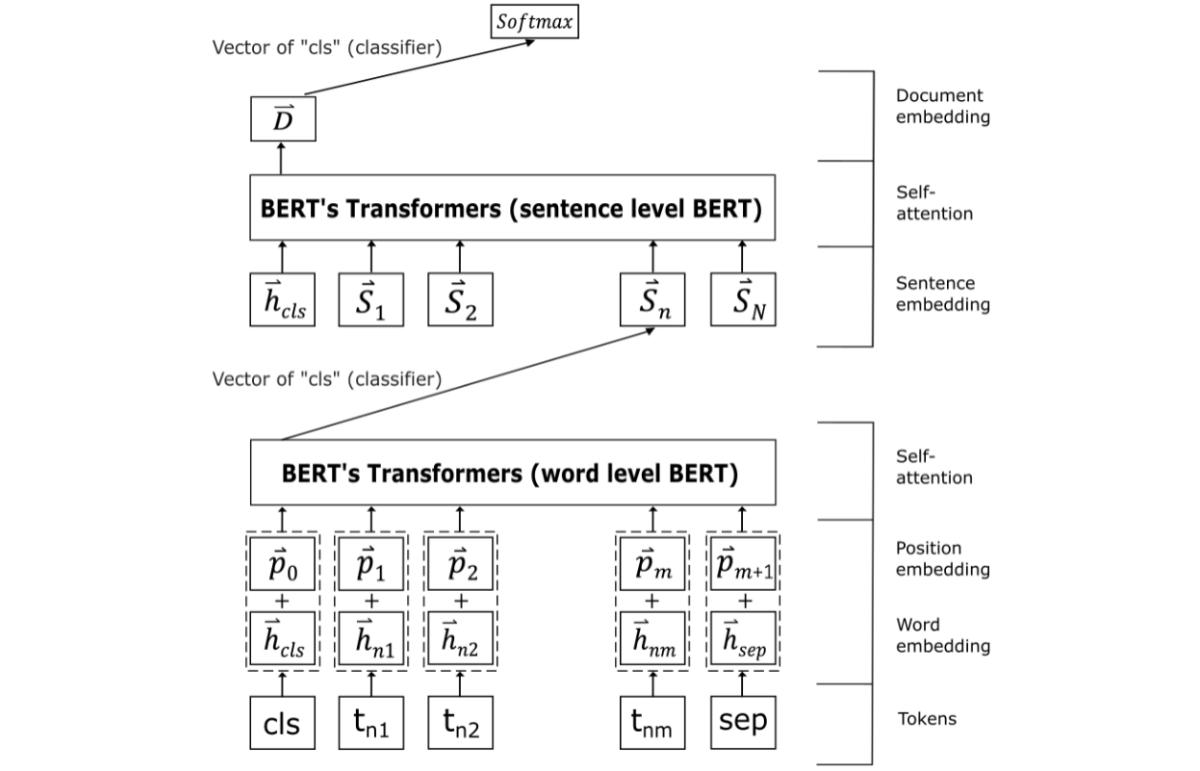
Composition of input sequence representations for text classification using BERT. The meaning of one sentence is summarized into the vector of [CLS] (classifier), an artifact token concatenated at the beginning of each sentence to become the sentence embedding. The sentence embedding is then fed to another transformer to generate the document embedding. An output layer with SoftMax activation provides the probability of text classification. BERT: bidirectional encoder representations from transformers.

A hierarchical attention network (HAN) structure with bidirectional encoder representations from transformers (BERT) was used to classify the text in the EMR clinical notes [33,34]. BERT is a contextualized word representation model that uses a masked language model that predicts randomly masked words in a context sequence. Publicly released BERT parameters are trained on corpora such as Wikipedia, which is formatted differently from clinical text. As such, ClinicalBERT, a language model specifically pretrained using clinical notes, was used for the text evaluation [35]. Medical language has been demonstrated to contain vast amounts of discipline-specific jargon, abbreviations, and acronyms while being a domain-specific and technical language [36]. Multiple studies have demonstrated that ClinicalBERT performs better than BERT [37,38]. Therefore, the decision to proceed with ClinicalBERT for our study was made. The ClinicalBERT embedding was not fine-tuned with clinical notes data due to a moderate sample size. Rather, the ClinicalBERT was downloaded and tested from a GitHub repo found in the study where Alzentzer et al observed performance improvements on three common clinical NLP tasks after training BERT models on clinical notes and discharge summaries [38,39]. The document embedding layer weights were not taken from ClinicalBERT. As the maximum sequence length of BERT limits it from handling text with more than 512 tokens, sentence embeddings generated by ClinicalBERT are fed to another transformer to obtain the “document embedding,” a highly abstracted vector capturing global information about the whole document [35,38]. HAPI status was classified based on this document embedding (Figure 4). The project-specific document embedding transformer was trained from the ground up through random initialization. Details of implementation and training of our HAN-BERT models are described in Figure S1 in Multimedia Appendix 1 [40,41].

### Model Evaluation

We used 5-fold stratified cross-validation to split the 97 positive cases and 183 patient controls into 5 groups. Due to the fact both numbers were not divisible by 5, there was a minor difference in the distribution of cases and controls between groups, although the effort was placed to retain the most similar distribution between the 5 groups. Each time we selected 4 groups as a training set, the remaining group was used as a test set. The splitting was the same for ML and deep learning experiments. A comparison of different document type subsets was executed with the best model to determine which document type subset would yield the best performance of PI detection. To fairly compare our method with ICD-based PI identification algorithms, the classification threshold was tuned to achieve similarly estimated specificities (0.988 and 0.959) of 2 ICD-based algorithms developed and validated in a previous study by Ho et al [1]. The first case definition is more specific and yields greater detection precision. The second definition is more inclusive of nonspecific codes for wounds and is likely to capture a larger number of cases. Sensitivity, specificity, positive predictive value (PPV), negative predictive value (NPV), and *F*_1_-scores were calculated as target metrics. Additionally, PPV, NPV, sensitivity, and specificity of the 4 algorithms and 2 ICD algorithms were calculated with changing thresholds ranging from 0.05 to 0.95. *F*_1_-score is a measure of accuracy through a combination of sensitivity and PPV. F1 has a maximum score of 1 when both sensitivity and PPV are 1, and a minimum of 0 when either sensitivity or PPV is 0. We calculated the binomial proportion CIs for sensitivity, specificity, PPV, and NPV using the Statsmodels package in Python (Python Software Foundation) [42]. The CIs of the *F*_1_-score were from 5-fold cross-validation.

### Ethics Approval

The study was approved by the Conjoint Health Research Ethics Board at the University of Calgary, Calgary, Alberta (REB13-0794).

## Results

### Characteristics of Study Cohort

The study included 280 eligible participants (Figure 2). Among the 280 patients, a research nurse identified 183 patients with no HAPIs, and 97 patients were found to have HAPIs. Table 1 provides demographic details of the patient cohort. The *P* values were calculated with MedCalc’s statistical calculators [43,44]. The median age was 68 (IQR 55-79) years. The cohort consisted of 127 (45.36%) females, and the median length of stay was 46 (IQR-79) days. A more detailed review of input data and linguistic inquiry and word count analysis for the number of words, sentences, and patients based on document types can be found in Table S1 in Multimedia Appendix 2.

**Table 1:** Descriptive statistics of patients (N=280).

Characteristics	All	Patients with pressure injury (n=97)	Patients without pressure injury (n=183)	*P* value
Female, n (%)	127 (45.36)	42 (43.30)	85 (46.45)	.62
Age (years), median (IQR)	68 (55-79)	68 (59-80)	67 (53-79)	.10
Length of stay (days), median (IQR)	46 (22-104)	48 (28-96)	46 (19-109)	.37
Cerebrovascular disease, n (%)	137 (48.93)	50 (51.55)	87 (47.54)	.52
Chronic obstructive pulmonary disease, n (%)	54 (19.29)	26 (26.80)	28 (15.30)	.02
Congestive heart failure, n (%)	66 (23.57)	30 (30.93)	36 (19.67)	.04
Myocardial infarction, n (%)	33 (11.79)	9 (9.28)	24 (13.11)	.34
Dementia, n (%)	27 (9.64)	10 (10.31)	17 (9.29)	.79
Peripheral vascular disease, n (%)	51 (18.21)	21 (21.65)	30 (16.39)	.28
Hemiplegia or paraplegia, n (%)	119 (42.50)	39 (40.21)	80 (43.72)	.57
Leukemia, n (%)	2 (0.71)	1 (1.03)	1 (0.55)	.62
Lymphoma, n (%)	4 (1.43)	2 (2.06)	2 (1.09)	.50
Peptic ulcer disease, n (%)	41 (14.64)	21 (21.65)	20 (10.93)	.02
Moderate or severe renal disease, n (%)	55 (19.64)	28 (28.87)	27 (14.75)	.005
Liver disease, n (%)	23 (8.21)	8 (8.25)	15 (8.20)	.99
Diabetes mellitus, n (%)	91 (32.50)	39 (40.21)	52 (28.42)	.045
Solid tumor, n (%)	50 (17.86)	19 (19.59)	31 (16.94)	.58
Connective tissue, n (%) disease	51 (18.21)	24 (24.74)	27 (14.75)	.04
History of smoking, n (%)	118 (42.14)	45 (46.39)	73 (39.89)	.30
Currently smoking, n (%)	37 (13.21)	11 (11.34)	26 (14.21)	.50
History of illicit drug use, n (%)	15 (5.36)	7 (7.22)	8 (4.37)	.32
Currently use illicit drugs, n (%)	8 (2.86)	3 (3.09)	5 (2.73)	.85

### Data Linkage and Extraction

Table 2 shows the patient and document count and document word count for the patients eligible for this study. Most PI-positive patients had “Patient assessment” document type (60 (61.86%) patients with HAPI versus 82 (44.81%) patients without HAPI), and patients in the negative groups predominantly had “Patient assessment Neuro.” However, patients from both groups had a similar amount of “Patient care” during the trial (Table 2).

**Table 2:** Characteristics of extracted documents, different components of nursing notes, and the average number of documents written by nurses.

Document type		All (N=280)	Patients with HAPI (n=97)	Patients without HAPI (n=183)
**Patient assessment**				
	Number of patients with this type of document, n (%)	142 (50.71)	60 (61.86)	82 (44.81)
	Number of notes per patient, median (IQR)	1.00 (0.00-17.00)	12.00 (0.00-18.00)	0.00 (0.00-16.00)
	Word count per note, median (IQR)	376.00 (306.00-430.00)	385.00 (311.00-441.00)	370.00 (303.00-421.00)
**Patient assessment neuro**				
	Number of patients with this type of document, n (%)	141 (50.36)	39 (40.21)	102 (55.74)
	Number of notes per patient, median (IQR)	2.50 (0.00-13.00)	0.00 (0.00-13.00)	8.00 (0.00-13.00)
	Word count per note, median (IQR)	428.00 (343.0-498.0)	424.00 (324.00-493.50)	430.00 (346.00-499.00)
**Patient care**				
	Number of patients with this type of document, n (%)	280 (100)	97 (100)	183(100)
	Number of notes per patient, median (IQR)	16.00 (13.00-21.20)	16.00 (13.00-19.00)	16.00 (13.00-23.50)
	Word count per note, median (IQR)	138.00 (72.00-179.00)	147.00 (91.00-185.00)	133.00 (66.00-176.00)

### Document Subset and Classification Models

Across a subset of document types and all tested classification techniques, the combination of Patient Assessment, Patient Assessment Neuro, and Patient Care yielded the highest outputs in terms of target metrics.

The TF-IDF vectorizer with RF classifier demonstrated the best performance in terms of sensitivity, PPV, and NPV when fixed at the specificity of 0.988 and 0.959 thresholds. The performance results are reported in Table 3.

**Figure 5:**
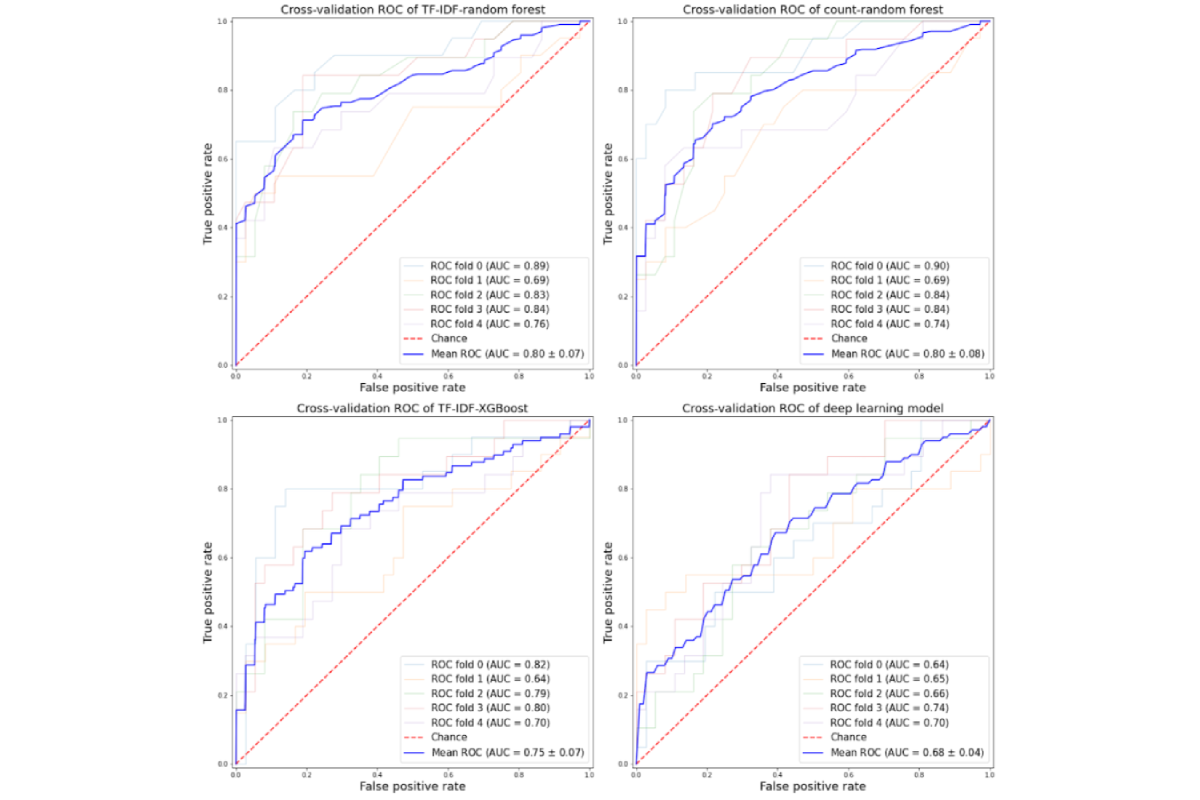
The ROC curves derived from the random forest with TF-IDF and word count, XGBoost, and deep learning models. AUC: area under the curve; ROC: receiver operating characteristic; TF-IDF: term frequency-inverse document frequency; XGBoost: eXtreme gradient boosting.

**Figure 6:**
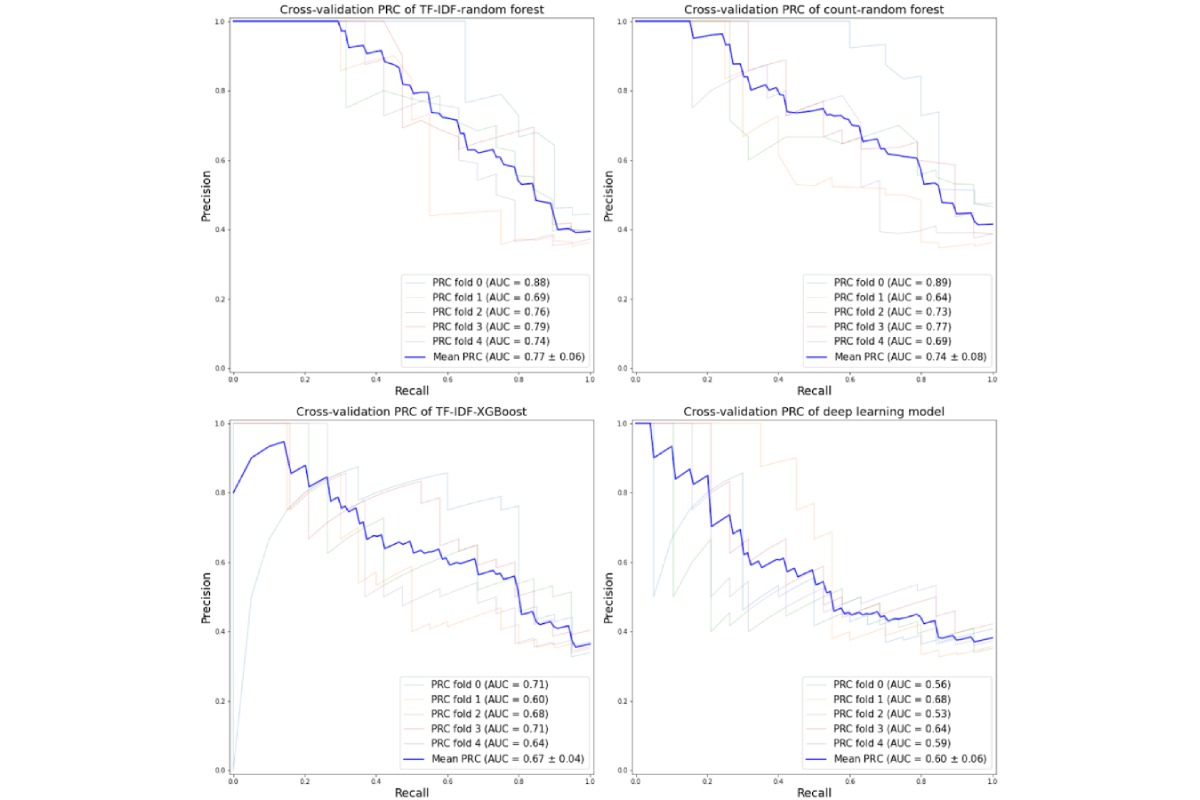
The area under the precision-recall curve (AU-PRC) performance of 4 models: random forest with TF-IDF and word count, XGBoost, deep learning model. AUC: area under the curve; PRC: precision-recall curve; TF-IDF: term frequency-inverse document frequency; XGBoost: eXtreme gradient boosting;

For a specificity of 0.988, the sensitivity of the (TF-IDF+RF) EMR-based model was 0.464 (95% CI 0.365-0.563), which surpassed the sensitivity 0.277 (95% CI 0.174-0.380) achieved by the ICD-based algorithm [1]. The PPV of our model had a mean of 0.938 (95% CI 0.869-1.000), which is higher than the reported 0.917 (95 % CI 0.854-0.980) of the ICD algorithm. The NPV was 0.776 (95% CI 0.722-0.830), which is also higher than the 0.739 (95% CI 0.638-0.840) reported in the ICD validation [1]. For a specificity of 0.959 achieved by the loose ICD-based algorithm, the EMR model sensitivity reached 0.546 (95% CI 0.447-0.645) compared to 0.328 (95% CI 0.220-0.436) found in ICD reporting [1]. Both PPV and NPV of EMR model were also higher (0.855 (95% CI 0.767-0.943) vs 0.793 (95% CI 0.700-0.886) and 0.798 (95% CI 0.745-0.851 vs 0.746 (95% CI 0.646-0.846) than those detected by ICD algorithm respectively. The deep learning model underperformed with the area under the receiver operating characteristic curve (AUC-ROC) score of 0.68 (SD 0.04), compared to the RF with the highest area under the curve (AUC) score of 0.80 (SD 0.08), followed by XGBoost with the AUC score of 0.75 (SD 0.07; Figure 5). Considering the prevalence of 34.6% in our study, the baseline area under the precision-recall curve (AU-PRC) is 0.346. Figure 6 shows that an AU-PRC of 0.77 (SD 0.06) was achieved for the RF models using TF-IDF tokenization, 0.74 (SD 0.08) was achieved for the RF models using count tokenization, 0.67 (SD 0.04) was achieved for the XGBoost models, and 0.60 (SD 0.06) for the deep learning models. These results are greater than 0.346, and we conclude that these classifiers do not discriminate by random chance and perform well in finding positive HAPI cases without accidentally marking negative patients as positive. Figure S1 in Multimedia Appendix 3 shows the PPV, NPV, sensitivity, and specificity of the 4 algorithms and 2 ICD algorithms, with changing thresholds ranging between 0.05 and 0.95.

**Table 3:** The performance of NLP^a^ methods on free-text electronic medical record documents at varying thresholds for the probability of pressure injury detection. The model was compared to ICD^b^ algorithms such that the model was trained to mimic its specificity.

Model		Sensitivity % (95% CI)	Specificity % (95% CI)	PPV^c^ % (95% CI)	NPV^d^ % (95% CI)	*F*_1_-score % (95% CI)
**Specificity near 0.988**						
	ICD (Ho et al [1])	0.277 (0.174-0.380)	0.988 (0.963-1.013)	0.917 (0.854-0.980)	0.739 (0.638-0.840)	0.425 (0.312-0.538)
	TF-IDF^e^+random forest^f^	0.464 (0.365-0.563)	0.984 (0.965-1.000)	0.938 (0.869-1.000)	0.776 (0.722-0.830)	0.612 (0.473-0.751)
	Count+random forest	0.412 (0.314-0.510)	0.978 (0.957-0.999)	0.909 (0.824-0.994)	0.758 (0.704-0.813)	0.550 (0.361-0.739)
	TF-IDF+XGBoost^g^	0.309 (0.217-0.401)	0.973 (0.949-0.996)	0.857 (0.741-0.973)	0.727 (0.671-0.782)	0.450 (0.340-0.559)
	Word Embedding+BERT^h^	0.268 (0.180-0.356)	0.978 (0.957-0.999)	0.867 (0.745-0.988)	0.716 (0.660-0.772)	0.394 (0.207-0.580)
**Specificity near 0.959**						
	ICD (Ho et al [1])	0.328 (0.220-0.436)	0.959 (0.913-0.100)	0.793 (0.700-0.886)	0.746 (0.646-0.846)	0.464 (0.350-0.578)
	TF-IDF+random forest	0.546 (0.447-0.645)	0.951 (0.919-0.982)	0.855 (0.767-0.943)	0.798 (0.745-0.851)	0.665 (0.577-0.753)
	Count+random forest	0.423 (0.324-0.521)	0.956 (0.927 -0.986)	0.837 (0.733-0.940)	0.758 (0.702-0.813)	0.546 (0.359-0.733)
	TF-IDF+XGBoost	0.423 (0.324-0.521)	0.956 (0.927-0.986)	0.837 (0.733-0.940)	0.758 (0.702-0.813)	0.552 (0.404-0.699)
	Word embedding+BERT	0.289 (0.198-0.379)	0.967 (0.941-0.993)	0.824 (0.695-0.952)	0.720 (0.663-0.776)	0.420 (0.280-0.560)

^a^NLP: natural language processing.

^b^ICD: International Classification of Diseases.

^c^PPV: positive predictive value.

^d^NPV: negative predictive value.

^e^TF-IDF: term frequency-inverse document frequency.

^f^The best model*.*

^g^XGBoost: extreme gradient boosting.

^h^BERT: bidirectional encoder representations from transformers.

## Discussion

### Principal Findings

Multiple methods were applied, and different combinations of clinical text were analyzed to determine the efficiency of NLP models in detecting HAPIs from nursing notes. The results of NLP models were compared with the ICD-based algorithm reported in the previous study [1]. An AUC of 0.80 (SD 0.07) of the ML model indicates fair accuracy in terms of produced sensitivity and specificity. The results demonstrate that different combinations of EMR data leverage NLP models to improve upon ICD-10–based HAPI case definitions. The TF-IDF with RF produced higher sensitivity at a strict specificity level. The satisfactory performance of ML models indicates that the free-text documented during hospitalization contains valuable information for HAPI detection. Developing algorithms using EMR data will facilitate the timely and accurate capture of HAPI incidences and measure the quality of nursing practice during patient hospitalization.

From the forward document type selection, we found that apart from the notes that directly document skin conditions in the patient assessment, the entries noting the patient’s consciousness, nutrition, and mobility were helpful in indicating HAPI. This makes clinical sense because the reduced level of consciousness, nutrition, and mobility are factors that may contribute to HAPI [45]. In addition, our findings align with several risk factors of the Braden Scale [46]. Given that many factors of the Braden Scale are documented in nursing notes daily, it may be promising to use NLP to automatically extract the Braden Scale’s factors and achieve better PI detection or prediction upon the automatically rated Braden Scale [46].

The results showed that the XGBoost and RF methods perform better than the advanced deep learning models by a large margin. The joint effort of the TF-IDF vectorizer and tree-based classifier enabled the pipeline to stay robust to irrelevant vocabularies, even when the sample size was smaller than the feature size. The feature selection played a role in this task because a great part of the text in clinical documents was not relevant to HAPIs and only contributed noise for the classification task. On the other hand, deep learning models allowed every input word to contribute to the document embedding upon which the model judged the presence of HAPIs. The suboptimal performance of the deep learning model may have been avoided if the transformers' attention mechanisms had more training samples to converge. The noisy data and not a very large sample size were possibly the main factors that made the deep learning models perform poorly. However, this hypothesis needs further examination.

Compared to these previous studies that used EMR to automate phenotyping, our model achieved higher sensitivity while reporting comparable values for performance metrics such as PPV and NPV. Furthermore, our model can identify HAPIs with high specificity and improved sensitivity during the first three days in routine clinical practice settings. Melton et al [47] found NLP to be reliable and effective in detecting 16 out of 65 adverse events in 1000 manually reviewed charts. The model by Melton et al [47] then processed all inpatient cases with EMR discharge summaries, achieving high specificity (0.9996) and low sensitivity (0.28). Our model results are in line with other studies that used free-text clinical notes to predict incidences of distinct adverse events [48-51].

### Limitations

The study is not without limitations. First, the exclusion of ICU patients due to data elements being distinct from SCM led to a smaller sample size and a narrower clinical cohort. Nevertheless, the remaining data from the clinical trial represented a population at risk for HAPIs. Second, both the patient and nurse knew at the admission of a clinical trial measuring PI would be the trial goal, which may have impacted the data entry quality of PI and frequency of patients to report PI-related discomfort. Third, the model produced relatively modest sensitivity. However, this sensitivity is deemed valuable, given that the specificity was set to a high threshold, and the input was restricted to the first 72 hours after enrollment [16]. The sensitivity reported in similar studies used the whole or more extended hospitalization stay and more data elements [50-52]. In addition, the sensitivity of our study was obtained through a comparison to a clinical trial instead of chart review data. Chart review does not always capture all positive cases due to possible errors in the review process [53,54]. Fourth, the comparison with deep learning is not likely to be very fair because BERT-based models are usually applied to larger cohorts. Nevertheless, our result can be served as a reference for model selection for researchers working with similar sample sizes. Prabhakar et al applied ClinicalBERT to phenotype 10 diseases on a cohort consisting of 1610 discharge summaries [41]. When only using ClinicalBERT, they obtained a very similar *F*_1_-score (0.46) compared to our result [41]. The suboptimal performances of the advanced deep learning model may suggest that study needs to be more evolved before applying deep learning to free-text-based clinical phenotyping. Tree-based ML models are recommended for detecting adverse event conditions from noisy, moderately sized text samples.

### Future Directions

The present work focused on demonstrating ML models on cross-sectional EMR data can outperform the ICD-based PI identification algorithm. Future directions could include (1) leveraging cost-sensitive learning to assign various weights to assess the impact of misclassifying the patients with a PI, (2) identification of the potential risk features or predictors that may be associated with PI, (3) comparison of HAN-BERT against other novel NN structures, and (4) detailed ablation studies for assessing the performance of components on the designed models that will hopefully be integrated into a clinical decision-support system. These studies will require larger sample sizes than our current pilot study, but our current work can be used to create such a cohort.

### Conclusions

Our study revealed the feasibility of using inpatient clinical notes documented for 3 days to detect HAPIs with increased accuracy over ICD methods. NLP and ML application on inpatient clinical notes allowed better and more timely use of the clinical narratives compared to summarizing them into ICD codes and DAD, thereby being a promising solution for precise, time-sensitive, population-based disease phenotyping. With the advent of digital technologies in health care, the results contribute toward an automated approach to better cohort identification, patient surveillance, and quality improvement for the treatment of hospital-acquired adverse events. The application of the model is particularly relevant for effectively mining clinical data that does not capture a large sample size for adverse effects phenotyping. The proposed method of identifying patients in acute care hospitals who are likely to have or develop PI will most likely be used by front-line hospital staff to prevent or manage PI earlier and more effectively.

## Appendix

Multimedia Appendix 1 Implementation details.

Multimedia Appendix 2 Linguistic inquiry and word count analysis.

Multimedia Appendix 3 Positive predictive value (PPV), negative predictive value (NPV), sensitivity, and specificity of the four algorithms and two International Classification of Diseases (ICD) algorithms, with changing thresholds ranging between 0.05 and 0.95.

## Abbreviations

AUC: area under the curve
AUC-ROC: area under the receiver operating characteristic curve
AU-PRC: area under the precision-recall curve
BERT: bidirectional encoder representations from transformers
BOW: bag-of-words
DAD: discharge abstract database
EMR: electronic medical record
HAN: hierarchical attention network
HAPIs: hospital-acquired pressure injuries
ICD: International Classification of Diseases
ICU: intensive care unit
ML: machine learning
NLP: natural language processing
NPV: negative predictive value
PI: pressure injury
PPV: positive predictive value
RCT: randomized controlled trial
RF: random forest
SCM: Sunrise Clinical Manager
TF-IDF: term frequency-inverse document frequency
XGBoost: extreme gradient boosting
