# Supplementary material for: Developing an Inpatient Electronic Medical Record Phenotype for Hospital-Acquired Pressure Injuries: Case Study Using Natural Language Processing Models
Source: JMIR AI. 2023 Mar 8;2:e41264. doi: 10.2196/41264 (PMC11041460; doi:10.2196/41264)
Supplement: Multimedia Appendix 1 [file ai_v2i1e41264_app1.docx]

Appendix 1: Implementation Details

The configuration of our ClinicalBERT model follows the original BERT base model except for the number of transformer layers. In particular, our model has 768 hidden states and 12 heads as the ClinicalBERT; the difference is that we used only the first 4 layers. The first 4 layers were chosen for the reasons below:

First, Liu et al. found the middle layers to be more suitable for transfer learning.[40]


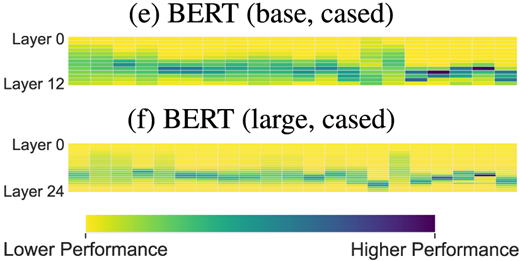


Figure S1 in Multimedia Appendix 1. A visualization of layerwise patterns in the task.[40] The middle layers often outperform the last layer for transfer learning.

Second, Prabhakar et al. conducted automated patient phenotyping experiments with 4 layers of ClinicalBERT and obtained satisfactory results.[41]

Third, we use GPUs (Nvidia Tesla v100) with 16G of memory for model development; thereby, we applied fewer layers to reduce model parameters.

The document sentence level BERT that uses sentence embedding instead of word embedding has one layer of transformer with 768 hidden states and 12 heads. A model with 2 layers of the transformer was tried and showed no improvement.

We used a learning rate of 0.00001(1e^-5^) for word-level BERT and 0.00005(5e^-5^) for sentence-level BERT. We let the model run 60 epochs and pick the best model based on its sensitivity on the test set when fixed the specificity. The best model typically appears before the 30^th^ epoch, and the sensitivity fluctuates afterwards.
