# Supplementary material for: Developing an Inpatient Electronic Medical Record Phenotype for Hospital-Acquired Pressure Injuries: Case Study Using Natural Language Processing Models
Source: JMIR AI. 2023 Mar 8;2:e41264. doi: 10.2196/41264 (PMC11041460; doi:10.2196/41264)
Supplement: Multimedia Appendix 2 [file ai_v2i1e41264_app2.docx]

Table S1. Linguistic inquiry and word count analysis for a comparison of number of words, sentences and patients based on document types.

| **Document type** | **#Words (STD)** | **#Sentences (STD)** | **#Patients** |
| --- | --- | --- | --- |
| Intake and Output | 24.48 (15.50) | 7.61 (3.59) | 280 |
| Clinical Record | 104.28 (62.35) | 3.72 (3.58) | 280 |
| Pain Assessment | 31.31 (15.70) | 2.68 (1.29) | 280 |
| MPR | 145.25 (127.13) | 11.82 (10.34) | 280 |
| Patient Care | 127.56 (60.22) | 21.7 (11.63) | 280 |
| Patient Assessment | 355.45 (122.43) | 54.95 (27.24) | 206 |
| Neurological Observation | 93.13 (16.67) | 15.34 (2.45) | 188 |
| Patient Assessment Neuro | 409.59 (134.11) | 62.71 (31.49) | 142 |
| Withdrawal - Alcohol Withdrawal Assessment | 88.45 (23.74) | 14.04 (6.33) | 141 |
| AcuityPlus Inpatient Classification | 68.38 (18.78) | 10.58 (1.64) | 140 |
| Patient Assessment Tools | 206.05 (67.26) | 18.85 (3.74) | 56 |
| Artificial Airway Record | 280.56 (71.00) | 31.53 (8.78) | 38 |
| Pharmacy Care Plan | 2160.84 (2187.81) | 117.92 (121.77) | 34 |
| Discharge Summary - Medical | 1812.21 (1150.32) | 129.47 (85.33) | 15 |
| Inpatient Operative/Procedure Report | 622.13 (294.43) | 43.47 (19.91) | 15 |
| PICC / Midline Record | 289.47 (88.28) | 49.73 (16.30) | 14 |
| Nursing Transfer Report - IP to IP | 668.00 (164.31) | 30.27 (17.14) | 12 |
| Social Work Assessment | 568.17 (180.22) | 37.17 (8.77) | 11 |
| Discharge Summary - Stroke Neurology | 714.09 (251.61) | 35.91 (17.61) | 8 |
| Suction - Airway | 17.70 (5.24) | 7.70 (1.62) | 8 |
| Neurological Diagnostics | 354.30 (68.81) | 30.40 (3.56) | 7 |
| History & Physical Examination. | 1247.62 (861.24) | 135.12 (46.25) | 7 |
| Inpatient Consult Report. | 1439.50 (679.27) | 106.12 (60.22) | 7 |
| EEG Preliminary Report | 83.00 (27.01) | 6.57 (3.66) | 4 |
| Palliative Care Consult | 925.71 (447.46) | 59.29 (43.21) | 4 |
| Discharge Summary - General | 1779.25 (614.54) | 145.00 (76.93) | 3 |
| ACP / GCD Tracking Record | 141.00 (21.23) | 6.33 (0.47) | 3 |
| Inpatient Consultation | 762.67 (28.02) | 86.67 (40.19) | 3 |
| ED UCC - Intake and Output | 8.00 (0.00) | 5.00 (0.00) | 2 |
| Therapy Assistant Assignment | 432.50 (141.50) | 22.00 (2.00) | 2 |
| Discharge Summary - Orthopedic Surgery | 956.50 (367.50) | 60.00 (14.00) | 1 |
| HoNOS (Adult) | 160.00 (0.00) | 26.00 (0.00) | 1 |
| Discharge Summary. | 854.00 (0.00) | 46.00 (0.00) | 1 |
| Therapist Assistant Assignment | 898.00 (0.00) | 79.00 (0.00) | 1 |
| Transfer Summary. | 1035.00 (0.00) | 61.00 (0.00) | 1 |
| ARO Hospital Admission Screen - Adult | 118.00 (0.00) | 6.00 (0.00) | 1 |
| Fiberoptic Endoscopic Evaluation of Swallowing | 534.00 (0.00) | 20.0 (0.00) | 1 |
