# Supplementary material for: Developing an Inpatient Electronic Medical Record Phenotype for Hospital-Acquired Pressure Injuries: Case Study Using Natural Language Processing Models
Source: JMIR AI. 2023 Mar 8;2:e41264. doi: 10.2196/41264 (PMC11041460; doi:10.2196/41264)
Supplement: Multimedia Appendix 3 [file ai_v2i1e41264_app3.docx]

Appendix 3: PPV, NPV, sensitivity, and specificity of the four algorithms and two ICD algorithms, with changing thresholds ranging between 0.05 to 0.95.

Figure S1 in Multimedia Appendix 3 shows the PPV, NPV, sensitivity, and specificity of the four algorithms and two ICD algorithms, with changing thresholds ranging between 0.05 to 0.95. The two dashed lines in each subfigure represent the 0.05 and 0.95 threshold bounds, respectively. TFIDF-RF represents the algorithm “TF-IDF + random forest”; WC-RF represents the algorithm “count + random forest”; WE-BERT represents the algorithm “word embedding + BERT”; ICD-1 and ICD-2 represent two ICD algorithms using the definition 1 and 2 in the algorithm by Ho et al., respectively.[1]


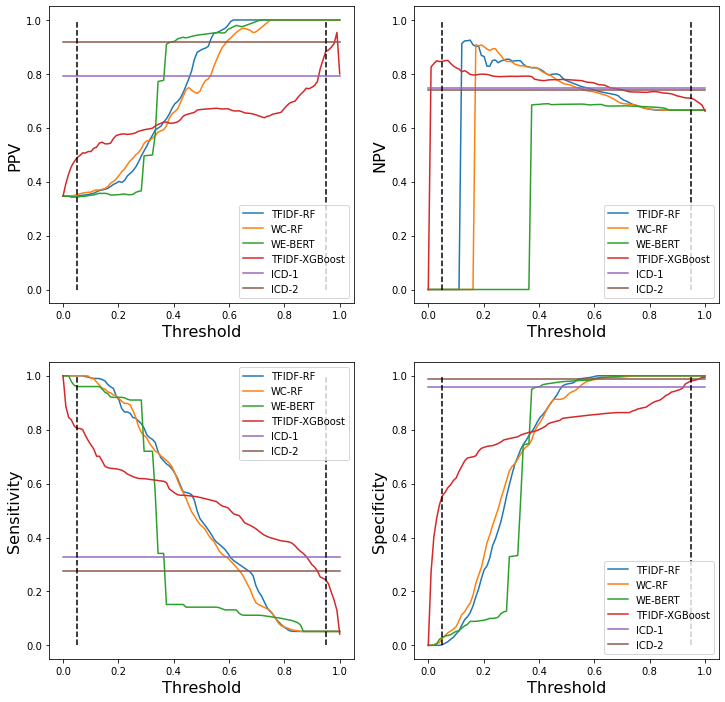


Figure S1 in Multimedia Appendix 3. PPV, NPV, sensitivity, and specificity of the four algorithms and two ICD algorithms, with changing thresholds ranging between 0.05 to 0.95.
